# STRA6 and Placental Retinoid Metabolism in Gestational Diabetes Mellitus

**DOI:** 10.3390/jpm11121301

**Published:** 2021-12-05

**Authors:** Arrigo Fruscalzo, Luigi Viola, Maria Orsaria, Stefania Marzinotto, Michela Bulfoni, Lorenza Driul, Ambrogio P. Londero, Laura Mariuzzi

**Affiliations:** 1 Clinic of Obstetrics and Gynecology, University Hospital of Fribourg, 1708 Fribourg, Switzerland; 2 Clinic of Radiology, University Federico II, 80138 Naples, Italy; luigiviola19@libero.it; 3 Institute of Clinical Pathology, University of Udine, 33100 Udine, Italy; mariaorsaria@yahoo.it (M.O.); stefania.marzinotto@uniud.it (S.M.); michela.bulfoni@uniud.it (M.B.); laura.mariuzzi@uniud.it (L.M.); 4 Clinic of Obstetrics and Gynecology, University of Udine, 33100 Udine, Italy; lorenza.driul@uniud.it (L.D.); ambrogio.londero@gmail.com (A.P.L.); 5 Ennergi Research (Non-Profit Organisation), 33050 Lestizza, Italy

**Keywords:** gestational diabetes mellitus, pregnancy, placenta, STRA6, vitamin A, retinoids, retinol-binding protein

## Abstract

Background: Recent reports indicate the potential role of the stimulated by retinoic acid 6 (STRA6) protein in developing insulin resistance. The study’s objective was to assess placental STRA6 expression and staining pattern in human pregnancy complicated by gestational diabetes mellitus (GDM). The expression pattern of further relevant genes involved in retinoid metabolism was also evaluated. Methods: A retrospective case–control study on paraffin-embedded placental tissue. Twenty-two human pregnancies affected by GDM, namely, 11 insulin-treated (iGDM) and 11 diet-controlled (dGDM), were compared with 22 normal-developed pregnancies (controls). An RT-PCR was performed in a random sample of 18 patients (six iGDM, six dGDM, and six controls) to assess RNA expression of STRA6 and further markers of retinoid metabolism. A semi-quantitative intensity evaluation at immunohistochemistry was performed for STRA6 in all 44 recruited patients. Results: STRA6 showed a decreased placental staining (9.09% vs. 68.18% positively stained samples, *p* < 0.05) and augmented RNA expression in dGDM patients than controls (ΔCT expression 0.473, IQR 0.403–0.566 vs. 0.149, IQR 0.092–0.276, *p* < 0.05). The protein staining pattern in patients affected by iGDM was comparable to controls. A reduced RNA expression of LPL, LRP1, VLDLR, and MTTP besides an augmented expression of LDLR was found in dGDM, while overexpression of LRP1 and LPL was found in iGDM patients. Unlike in the control group, significant positive correlations were found between RXRα and the proteins involved in the intracellular uptake of ROH, such as STRA6, LRP1, LRP2, and VLDLR. Conclusions: An altered placental expression and staining pattern of STRA6 were found in pregnancies complicated by GDM compared to the controls. These changes were coupled to an altered expression pattern of several other genes involved in the retinoid metabolism.

## 1. Introduction

Gestational diabetes mellitus (GDM) is a pregnancy-related pathology defined as glucose intolerance first diagnosed during pregnancy and disappearing after delivery [1]. It affects about 7% of all pregnancies, with an increasing incidence worldwide, making it a major medical issue [2]. GDM is responsible for important obstetric complications during pregnancy and delivery (such as polyhydramnios, fetal macrosomia, and stillbirth), as well as during the neonatal period (e.g., hypoglycemia, hyperbilirubinemia, polycythemia, cardiomyopathy, and respiratory distress) [3]. Similar to diabetes mellitus type 2, hyperglycemia in the GDM is linked to a relatively inadequate insulin secretion due to insulin resistance [4]. Physiopathology of GDM is multifactorial, with both genetic and complex environmental factors being implicated [5,6].

Retinol plays key roles in DNA expression and cell fate determination, resulting in being essential in the development of the placenta and the fetus [7]. Past research has evidenced its importance, together with its transport complex transthyretin–retinol-binding protein (TTR-RBP4), in developing several pregnancy complications [8,9,10,11,12]. ROH can be transported either within target cells, such as trophoblast cells, by a stimulated by retinoic acid 6 (STRA6) protein-mediated pathway that specializes in the uptake of ROH transported by the TTR, RBP4, and ROH complex, or by a pathway independent of STRA6 in the form of esters of the ROH transported within the cytosol in a non-specific way through the absorption of chylomicrons and lipoproteins [13]. Several membrane receptors contribute to this alternative and non-specific pathway, including VLDLR, LDLR, LRP1, and LRP2 [13].

Studies in animal models have also shown how a partial deficiency of the trans-membrane receptor STRA6, and therefore of the intracellular uptake of ROH mediated by it can be compensated by the alternative routes of ROH transport to ensure adequate dietary vitamin A support [13,14,15]. Wassef et al. highlight a particular role of LRP1 in mediating the uptake of β-carotene through the placenta and show its activation in response to chronic changes in the vitamin A content in the maternal diet, both in terms of supplementation with β-carotene (which determines decreased transcription of LRP1) and in the case of mild vitamin A deficiency (which induces the uptake of β-carotene) [16].

Great interest has been focused on the retinol-binding protein–retinol complex (RBP4-ROH) and STRA6, its transmembrane receptor [13]. Recent research indicates, indeed, that STRA6 is not only responsible for ROH intake but also, through the binding of RBP4 and ROH, can be involved in the onset of insulin resistance through the activation of the JAK/STAT pathway [17]. However, the role played by STRA6 in pregnancies complicated by insulin resistance has not been explored yet, and uncertainty remains regarding the underlying mechanisms potentially involved.

This study’s objective was to evaluate the placental expression and staining pattern of STRA6 and the expression pattern of some of the most relevant enzymes involved in the metabolic pathway of retinoids during gestational diabetes.

## 2. Materials and Methods

### 2.1. Design, Setting, and Study Population

This retrospective case–control study was conducted on placental tissue collected between 2014 and 2016 at the University Hospital in Udine. Included were 22 singleton pregnancies affected by gestational diabetes mellitus, namely, 11 insulin-treated (iGDM) and 11 diet only-treated (dGDM) compared with 22 normal developed pregnancies as controls. The clinical information concerning the pregnancies was collected from the archives of the Women’s and Children’s Health Department of the University Hospital of Udine. Exclusion criteria were the following: preterm delivery, presence of concomitant pre-gestational or gestational maternal or fetal pathologies, pregnancies complicated by viral or bacterial infection, cigarette smoking during pregnancy, and known fetal chromosomal abnormalities. The internal review board approved the present study that was conducted following the Declaration of Helsinki. Moreover, this study attended the dictates of the general authorization to process personal data for scientific research purposes by the Italian Data Protection Authority.

### 2.2. Clinical Data

The pregnancy characteristics and outcomes were gathered retrospectively from medical records. The following data were retrieved: maternal age at delivery, pre-pregnancy body mass index (BMI), gestational age at delivery, parity, mode of delivery, Apgar score (at first and fifth minute), neonatal sex, neonatal weight, the prevalence of small for gestational age (SGA) newborns, the prevalence of large for gestational age (LGA) newborns, and the placental weight. This study set SGA as neonatal weight under the 10th percentile and LGA as neonatal weight over the 90th percentile for gestational age [18]. Furthermore, gestational diabetes was diagnosed through the 75 g two-hour oral glucose tolerance test according to the presence of risk factors at 16–18 or 24–28 weeks gestation [18].

### 2.3. Real-Time PCR

Real-time PCR was performed in a random sample of 18 patients (6 iGDM, 6 dGDM, and 6 controls) of the population studied. For each group, a randomly allocated sequence without replacement was extracted. The RNA extraction from placental tissue samples fixed in formalin and embedded in paraffin was carried out using the Qiagen RNeasy Mini Kit, which allows RNA to be isolated even from modest quantities of fixed tissue and embedded in paraffin. The kit was used according to the manufacturer’s instructions. Then, the RNA obtained was back-transcribed to cDNA using the “SuperScript III REV” reverse transcriptase (Waltham, MA, USA). A quantitative RT-PCR reaction was then carried out using the Roche LightCycler 480 system (Roche, Basel, Switzerland) and the SSOADV UNIVER SYBR GRN SMX 500 (BIO-RAD, Hercules, CA, USA), according to the manufacturer’s protocols. Using the primers for STRA6, LPL, LRP1, LRP2, LDLR, VLDLR, MTTP, RXRα, and RXRβ, we performed the reactions in triplicate in three independent experiments (Table S1). The quantification of mRNA was expressed in terms of the threshold cycle (CT). The average value of the CT was obtained from the triplicates, which were used for the subsequent analyses. All assays expressed in CT were normalized concerning the CT value of glyceraldehyde-3-phosphate dehydrogenase (GAPDH). The differences between the CT values of the tested genes and those of the reference genes were calculated as ΔCT(gene) = CT(gene) − CT(GAPDH).

### 2.4. Immunohistochemistry

Immunohistochemistry was performed on all 44 patients studied (11 iGDM, 11 dGDM, and 22 controls). From the paraffin blocks containing the placental tissue, we obtained 4 µm thick cross-sections. Then, after placing the section on a slide, they were deparaffinized, rehydrated, and incubated with H_2_O_2_ at 3% for 10 min to block the endogenous peroxidase activity. The antigen was later unmasked in a citrate buffer at pH 6 and at a temperature of 98 °C. The same sections were then washed in PBS and incubated in a humid chamber at room temperature for 60 min with STRA6 antibody diluted at 1:50 (Abcam, Cambridge, MA, USA). We then incubated the samples with Dako REAL EnVision/HRP, Rabbit/Mouse reagent for 30 min at room temperature. The Dako REALE™ DAB + Chromogenic Substrate (Dako, Glostrup, Denmark), used in accordance with the manufacturer’s instructions, was used to detect peroxidase (HRP) activity. Finally, the sections were contrasted with Gil’s Hematoxylin prior to mounting and observing under the microscope.

The immunohistochemical data were presented as semi-quantitative scores. Cytoplasmic and membrane staining was recorded as an intensity score, where staining intensity was evaluated using a three-grade score: 3 for strong, 2 for moderate, and 1 for weak. Nuclear staining data were presented as H-score, ranging between 0 and 300. The H-score was calculated as the product between the percentage of cell nuclei positive for staining and the score given to the staining intensity ranging from 3 (strong) to 1 (weak). 

### 2.5. Statistical Analysis

R v3.2.3 (R Foundation for Statistical Computing, Vienna 2015) was used for data analysis in this study. A *p*-value less than 0.05 (*p* < 0.05) was considered significant. RT-PCR data were presented as ΔCT expression (i.e., 2^(−ΔCT)^). The Kolmogorov–Smirnov test evaluated the normality of the distribution of continuous variables. The text reports continuous parametric distributions as mean (±standard deviation) and non-parametric distributions as median and interquartile ranges (IQR). Categorical values were presented as percentage and absolute values. The differences between groups were evaluated with the *t*-test (Student’s *t*) for variables with a normal distribution. For variables with a non-parametric distribution, the Wilcoxon test was used. Furthermore, the correlations between the variables considered were evaluated employing the Spearman test. Where appropriate, differences between groups were assessed for categorical variables, with the chi-squared test or Fisher’s exact test.

## 3. Results

The characteristics of the population studied by immunohistochemistry and RT-PCR analysis are described in Table 1A,B. All the patients considered in this study were Caucasian. The maternal age was 35.0 years (IQR 30.0–37.5) in the dGDM, 36.0 years (32.0–37.5) in the iGDM, and 32.0 years (IQR 30.2–33.0) in the control group (*p* > 0.05) (Table 1A). The pre-pregnancy BMI was significantly higher in the iGDM than in the dGDM group (*p* < 0.05) (Table 1A). All the cesarean sections among the included cases were due to repeated cesarean section or labor induction failure, and non-differences were observed among the three groups. Although this study found no differences in the weights of infants, there was a higher incidence of LGA and a significantly higher placental weight in the iGDM group than in other groups (Table 1A). No significant differences were observed in the characteristics of the population undergoing RT-PCR analysis (Table 1B). Overall, we did not see any further significant differences in delivery type or parity, the sex of the newborn, and the Apgar score at the first and fifth minute after birth (Table 1A,B).

The RT-PCR analysis of the placental tissue is reported in ΔCT expression. In the control group, there was a reduced expression of LPL and LRP1 compared to the iGDM group (*p* < 0.05) (Table 2). Furthermore, considering the dGDM, this group showed a reduced level of expression of all the proteins studied except STRA6 and LDLR, which was expressed at a significantly increased level compared to both controls and iGDM (*p* < 0.05) (Table 2).

**Figure 1:**
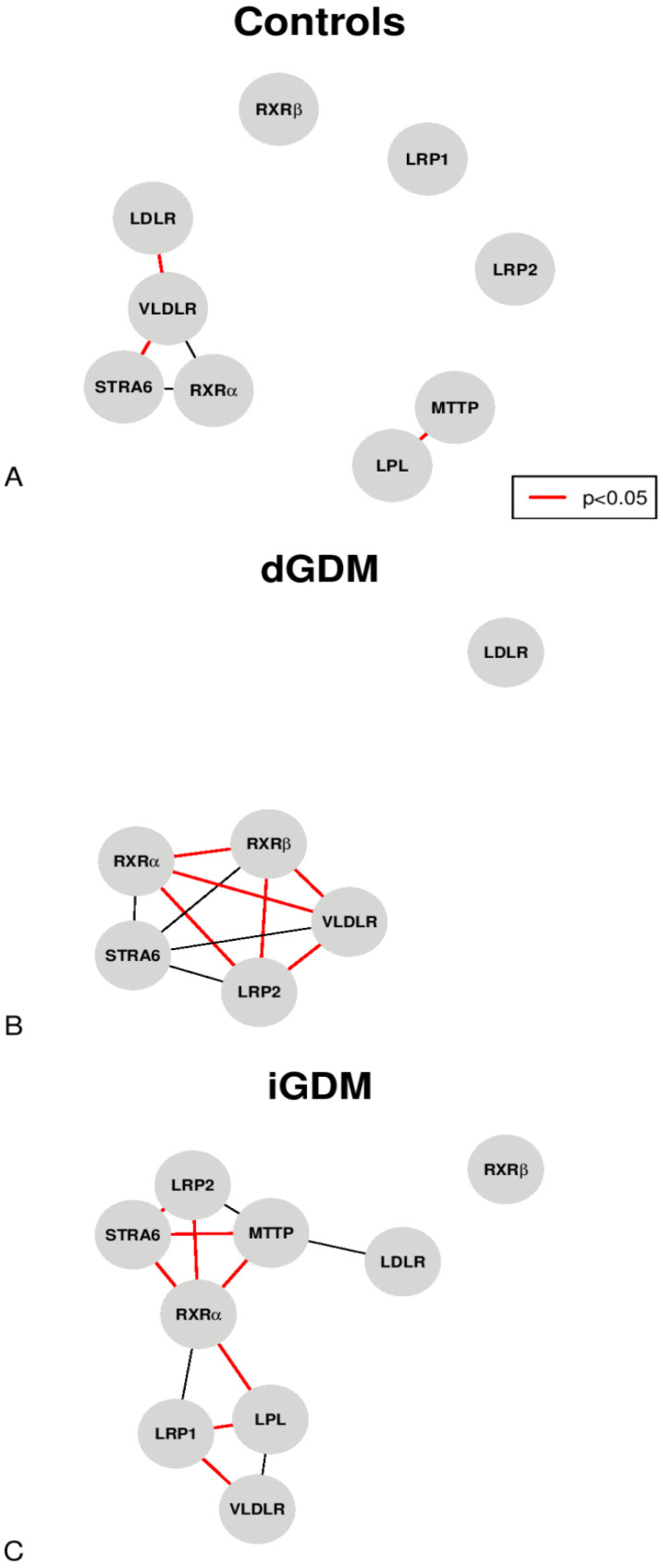
Analysis of the correlation network with an absolute value of rho > 0.70 (the red lines represent the significant correlations *p* < 0.05): (**A**) in the control group; (**B**) in the dGDM group; (**C**) in the iGDM group.

Figure 1A–C shows the correlations, with an absolute value of rho > 0.70, between the various proteins analyzed by RT-PCR in the three study groups. In the group of controls, LPL and MTTP were significantly and positively correlated (rho = 0.81, *p* < 0.05); moreover, STRA6 and VLDLR were also positively correlated (rho = 0.89, *p* < 0.05). Furthermore, LDLR and VLDLR were inversely correlated (rho = −0.75, *p* = 0.084) (Figure 1A). Within the dGDM group (Figure 1B), RXRα was positively correlated to RXRβ (rho = 1.00, *p* < 0.05), VLDLR (rho = 1.00, *p* < 0.05), and LRP2 (rho = 1.00, *p* < 0.05); moreover, RXRβ was significantly correlated with VLDLR (rho = 1.00 *p* < 0.05) and LRP2 (rho = 1.00, *p* < 0.05). In addition, LRP2 was positively correlated to VLDLR (rho = 1.00, *p* < 0.05). In the iGDM group (Figure 1C), RXRα was positively correlated with STRA6 (rho = 0.89, *p* < 0.05), LRP1 (rho = 0.74, *p* = 0.057), and LRP2 (rho = 0.82, *p* < 0.05), while it was negatively correlated with LPL (rho = −0.82, *p* < 0.05) and MTTP (rho = −0.82, *p* < 0.05). Furthermore, LPL was significantly and negatively correlated to LRP1 (rho = −0.93, *p* < 0.05). LRP1 was positively correlated to VLDLR (rho = 0.90, *p* < 0.05). In addition, LRP2 was negatively correlated with MTTP (rho = −0.74, *p* = 0.057) and positively correlated with STRA6 (rho = 0.86, *p* < 0.05). Finally, STRA6 was negatively correlated with MTTP (rho = −0.89, *p* < 0.05).

**Figure 2:**
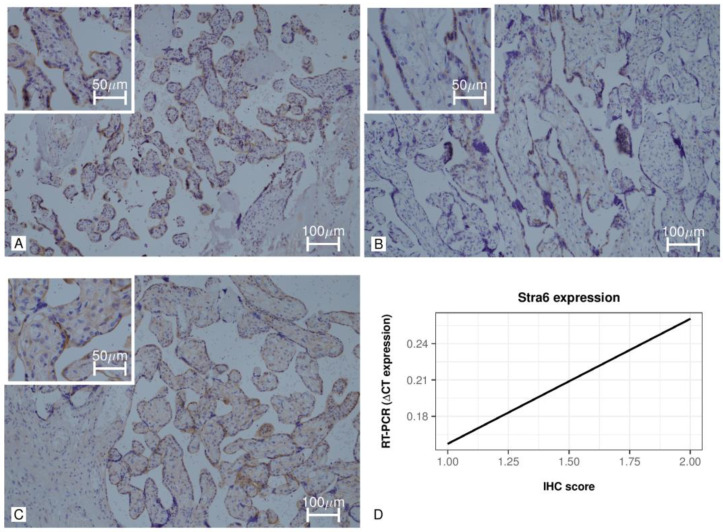
Placental immunohistochemical staining of STRA6. (**A**) Image at 20× (and in the box at 40×) of STRA6 placental immunohistochemical staining in a subject of the control group. (**B**) Image at 20× (and in the box at 40×) of STRA6 placental immunohistochemical staining in a subject of the dGDM group. (**C**) 20× image (and in the 40× square) of STRA6 placental immunohistochemical staining in a subject of the iGDM group. (**D**) Correlation between RT-PCR ΔCT expression and IHC score.

The immunohistochemical analysis of STRA6 is reported in Table 3 and Figure 2A–C. STRA6 resulted in immunohistochemical staining mainly localized to the cytoplasm and the cytoplasmic membrane. Moreover, the STRA6 immunohistochemical staining resulted in a patchy or ’mosaic’ pattern. No significant differences were found between the three groups considering the cytoplasmic immunohistochemical score; however, the cytoplasmic membrane was less frequently positive in the dGDM group than the iGDM (*p* = 0.061) and controls (*p* < 0.05) (Figure 2A–C). Furthermore, the percentage of positive villi was higher in controls and iGDM groups than in the dGDM group. Finally, a non-significant positive correlation was found between immunohistochemical staining and ΔCT expression (rho = 0.414, *p* = 0.414) (Figure 2D).

## 4. Discussion

In this study, we explored the pathway of retinoid metabolism in human placentas with particular attention to the mechanisms involved in the cellular uptake of ROH itself. Here, we showed that women affected by GDM displayed a characteristic expression pattern of STRA6 and other placental markers of retinoid metabolism compared to controls. STRA6 showed a decreased placental staining at immunohistochemistry and augmented RNA expression in patients with dGDM. Interestingly, this expression pattern was comparable to controls in patients affected by iGDM. Moreover, further markers of retinoid metabolism were found to show a peculiar pattern. Here, a reduced expression of LPL, LRP1, VLDLR, and MTTP combined by an augmented expression of LDLR was found in dGDM, while overexpression of LRP1 and LPL was found in iGDM patients. Finally, unlike in the control group, significant positive correlations were found between RXRα and the proteins involved in the intracellular uptake of ROH, such as STRA6, LRP1, LRP2, and VLDLR. 

Our results highlight an increased expression of LRP1 and LPL in placentas of insulin-treated gestational diabetes and, in turn, an increase in the intracellular ROH apparatus by the non-STRA6 pathway. At the same time, we observed a significant inversely proportional correlation between LRP1 and LPL as well as between RXRα and LPL or MTTP. This result could represent an increased intake of ROH esters and possible negative feedback on this extrinsic pathway to limit an excessive intake of potentially toxic ROH esters. According to our data, we had in the iGDM and dGDM groups, unlike the controls, significant positive correlations between RXRα and the genes involved in the intracellular uptake of ROH such as STRA6, LRP1, LRP2, and VLDLR. This effect suggests a particular activity of this pathway not highlighted in the controls. 

Moreover, we found both a reduced synthesis of MTTP in dGDMs and an inversely proportional correlation between the transmembrane transporters of ROH and MTTP in iGDM. MTTP has a fundamental role in forming chylomicrons but, above all, VLDL [19]. VLDLs are produced at the trophoblast level to transfer lipids to the fetal circulation [20]. Since the VLDLs are transporters of ROH esters in the fetal circulation, a reduction in MTTP can be read as a protective mechanism against a possible increase in toxic levels of ROH in the fetal circulation. 

Furthermore, we observed an increased expression of membrane receptors involved, above all, in the independent STRA6 uptake of ROH. This finding could underpin an adaptation response to what has been observed in a previous work of our group, i.e., the reduction of ROH–RBP4–TTR in maternal serum during the first trimester of pregnancy of patients developing an iGDM [21]. Few studies have evaluated the relationship between serum ROH concentration and GDM [21,22,23]. Moreover, in previous studies carried out in non-pregnant women with insulin-dependent diabetes mellitus, a reduction in ROH concentration was demonstrated compared to controls, thus endorsing the results we present [21,24,25].

Still, in experiments on animal models, the STRA6 expression was identified at the level of the labyrinthine area of the mouse chorioallantoic placenta (the area dedicated to the exchange of nutrients), and the modulation of the expression of target genes was also demonstrated by RAR and RXR [26,27]. In addition, the induction of the retinoic acid membrane receptor STRA6 was found to be mediated preferentially by RXRα/RARγ heterodimers [26,27,28]. Thus, the role of STRA6 in placental development appears crucial, and it is suggested that STRA6 is itself a direct target of nuclear retinoid receptors. Our data also show correlations between STRA6 and RXR that were particularly significant in the groups affected by GDM treated with insulin. 

STRA6, in addition to being the transmembrane transporter for the ROH into the cytoplasm, has been discovered to have other functions. Berry et al. in 2012 assigned STRA6 a role in the intracellular signaling of circulating RBP4 [29]. In fact, the authors demonstrated that TTR blocks the association of holo-RBP4 with STRA6 so that the receptor would only function in circumstances in which the serum levels of RBP4 would exceed those of TTR, activating the signaling cascade culminating in the induction of STAT genes. Therefore, changes in the serum levels of RBP4 (or TTR) would alter the RBP4/TTR ratio, favoring the activation of signaling mediated by STRA6 [29]. Furthermore, a correlation between the serum levels of insulin and those of RBP4 has been described in the literature, suggesting that within a certain limit, insulin could cause an increase in the concentration of RBP4 [28]. Therefore, a state of hyperinsulinemia, as occurs during insulin resistance, determines an increase in the levels of RBP4 [30]. All of this can, in turn, modify the functionality of the ROH pathway. 

Notably, the inverse relationship between augmented STRA6 RNA expression levels and reduced staining at immunohistochemistry in dGDM could be explained by an augmented turnover of STRA6, functioning as a transmembrane receptor for the TTR–ROH–RBP4 complex. To date, we have no proven explanation for this data, but this hypothesis could be supported by a similar mechanism of regulation used by other transmembrane receptors like the insulin ones [31]. 

The study has some limitations related to its retrospective nature and the small sample size that analyzes the expression of proteins by RT-PCR. Moreover, paraffin-embedded tissue is not the best medium for performing RT-PCR, as the amount of available RNA could be reduced compared to the original. This limit could have affected the sensitivity of some less expressed genes studied. Nonetheless, we expect comparable differences in genes expression among the three categories of patients examined, at least for the most expressed ones. 

Moreover, some variables were not considered in the study, such as the intake of retinoids and carotenoids in the diet and other pathways of retinoid metabolism, which could potentially impact STRA6 activity. Furthermore, evaluating STRA6 and the metabolism of retinoids at the end of pregnancy does not allow for the translation of results to the whole pregnancy. These circumstances limit the possibility of obtaining translatable results in obstetric practice and proposing concrete therapeutic strategies yet to be applied. Nonetheless, the data obtained could help direct future investigations to improve the knowledge of disease pathogenesis and define new approaches to precision medicine.

## 5. Conclusions

In summary, this study revealed an altered expression and staining pattern of STRA6 in the placental tissue of the pregnancies affected by GDM compared to the controls. These changes were coupled to an altered expression pattern of other genes involved in the retinoid metabolism. These findings seem to indicate an impairment of the retinoid pathway in the context of this common pathology of pregnancy. Further insights into STRA6 and retinoid metabolism will hopefully lead to future clinical applications.

## Figures and Tables

**Table 1:** Population description. (**A**) Population description of the subjects assessed for STRA6 IHC. (**B**) Population description of the subjects assessed for RT-PCR.

**(A)**	**Controls (22)**	**dGDM (11)**	**iGDM (11)**	***p***
Maternal age (years)	32.0 (30.2–33.0)	35.0 (30.0–37.5)	36.0 (32.0–37.5)	NS
BMI (kg/m^2^)	23.00 (21.50–24.00)	20.05 (19.12–23.55)	24.90 (23.00–31.25)	3
Nulliparity	59.1% (13/22)	45.5% (5/11)	54.5% (6/11)	NS
Mode of delivery				
Vaginal birth	59.1% (13/22)	63.6% (7/11)	36.4% (4/11)	NS
Cesarean section	40.9% (9/22)	36.4% (4/11)	63.6% (7/11)	NS
Gestational age at delivery (weeks)	39.0 (38.0–40.0)	39.0 (37.5–40.0)	38.0 (38.0–38.0)	2
Neonatal sex (male)	40.9% (9/22)	36.4% (4/11)	45.5% (5/11)	NS
Apgar score first minute	8.0 (6.0–8.8)	9.0 (8.0–9.0)	9.0 (8.0–9.0)	NS
Apgar score fifth minute	9.0 (9.0–9.0)	9.0 (9.0–10.0)	9.0 (9.0–9.0)	NS
Neonatal weight (grams)	3351.0 (3048.0–3580.0)	3240.0 (3131.5–3376.5)	3214.0 (2942.5–3718.5)	NS
SGA (<10th centile)	0.0% (0/22)	18.2% (2/11)	9.1% (1/11)	NS
LGA (>90th centile)	0.0% (0/22)	9.1% (1/11)	27.3% (3/11)	2
Placental weight (grams)	595.00 (520.00–670.00)	582.50 (465.00–635.00)	700.00 (616.25–770.00)	2, 3
**(B)**	**Controls (6)**	**dGDM (6)**	**iGDM (6)**	***p***
Maternal age (years)	32.5 (32.0–36.8)	32.0 (27.5–36.5)	33.0 (29.8–35.5)	NS
BMI (kg/m^2^)	21.50 (21.25–21.75)	20.05 (19.55–21.52)	26.00 (25.00–33.00)	NS
Nulliparity	66.7% (4/6)	50.0% (3/6)	66.7% (4/6)	NS
Mode of delivery				
Vaginal birth	33.3% (2/6)	66.7% (4/6)	33.3% (2/6)	NS
Cesarean section	66.7% (4/6)	33.3% (2/6)	66.7% (4/6)	NS
Gestational age at delivery (weeks)	39.5 (38.2–40.0)	38.5 (37.2–39.8)	38.0 (38.0–38.0)	NS
Neonatal sex (male)	16.7% (1/6)	50.0% (3/6)	66.7% (4/6)	NS
Apgar score first minute	8.0 (8.0–8.0)	8.5 (8.0–9.0)	8.5 (8.0–9.0)	NS
Apgar score fifth minute	9.0 (9.0–9.0)	9.0 (9.0–9.0)	9.0 (9.0–9.0)	NS
Neonatal weight (grams)	3152.0 (2852.5–3540.0)	3376.5 (3337.5–3567.0)	3167.0 (2841.0–4028.5)	NS
SGA (<10th centile)	0.0% (0/6)	16.7% (1/6)	16.7% (1/6)	NS
LGA (>90th centile)	0.0% (0/6)	16.7% (1/6)	33.3% (2/6)	NS
Placental weight (grams)	522.50 (493.75–581.25)	615.00 (415.00–640.00)	650.00 (570.00–730.00)	NS

Acronyms: GDM = gestational diabetes mellitus, dGDM = gestational diabetes mellitus treated with diet alone, iGDM = gestational diabetes mellitus treated with insulin, BMI = body mass index, SGA = small for gestational age, LGA = large for gestational age. Significant differences (*p* < 0.05) between: (1) controls and dGDM; (2) controls and iGDM; (3) dGDM and iGDM.

**Table 2:** Results of RT-PCR analysis of formalin-fixed placental tissue considering proteins related to the retinoid pathway. Values are shown in ΔCT expression.

	Controls (6)	dGDM (6)	iGDM (6)	*p*
LPL	0.040 (0.022–0.048)	0.000 (0.000–0.000)	0.061 (0.044–0.085)	1, 2, 3
LRP1	0.018 (0.017–0.019)	0.000 (0.000–0.000)	0.043 (0.043–0.056)	1, 2, 3
LRP2	0.037 (0.019–0.069)	0.000 (0.000–0.000)	0.080 (0.034–0.086)	1, 3
MTTP	0.002 (0.001–0.004)	0.000 (0.000–0.000)	0.003 (0.002–0.005)	1, 3
STRA6	0.149 (0.092–0.276)	0.473 (0.403–0.566)	0.167 (0.116–0.279)	1, 3
RXRα	0.010 (0.005–0.018)	0.000 (0.000–0.000)	0.017 (0.011–0.020)	1, 3
RXRβ	0.001 (0.001–0.002)	0.000 (0.000–0.000)	0.002 (0.000–0.003)	1
LDLR	0.002 (0.001–0.004)	0.077 (0.071–0.097)	0.006 (0.001–0.009)	1, 3
VLDLR	0.004 (0.002–0.005)	0.000 (0.000–0.000)	0.005 (0.003–0.010)	1, 3

Acronyms: GDM = gestational diabetes mellitus, dGDM = gestational diabetes mellitus treated with diet alone, iGDM = gestational diabetes mellitus treated with insulin. Significant differences (*p* < 0.05) between: (1) controls and dGDM; (2) controls and iGDM; (3) dGDM and iGDM.

**Table 3:** Analysis of STRA6 staining at immunohistochemistry in placental tissue (the reported values are median and interquartile range (IQR), and the *p*-values are from the Wilcoxon test).

	Controls (22)	dGDM (11)	iGDM (11)	*p*
Cytoplasm (intensity score)	1.00 (1.00–2.00)	1.00 (0.50–1.50)	1.00 (1.00–1.50)	NS
Cytoplasmic membrane (intensity score)	1.00 (0.00–2.00)	0.00 (0.00–0.00)	0.00 (0.00–1.00)	1
Cytoplasmic membrane (positivity)	68.18% (15/22)	9.09% (1/11)	27.27% (3/11)	1
Villi (% positivity)	27.50 (11.25–35.00)	10.00 (2.50–18.75)	20.00 (5.00–42.50)	NS

Acronyms: GDM = gestational diabetes mellitus, dGDM = gestational diabetes mellitus treated with diet alone, iGDM = gestational diabetes mellitus treated with insulin. Significant differences (*p* < 0.05) between: (1) controls and dGDM; (2) controls and iGDM; (3) dGDM and iGDM.

## Data Availability

The data that support the findings of this study are available, but restrictions apply to the availability of these data, which were used under license for the current study, and thus are not publicly available. Data are, however, available from the authors upon reasonable request and with permission of the Internal Review Board.

## Supplementary Materials

The following are available online at https://www.mdpi.com/article/10.3390/jpm11121301/s1, Table S1: Here the primer sequences used are listed.

## Abbreviations

cDNA: complementary deoxyribonucleic acid; CT: threshold cycle; dGDM: diet-controlled gestational diabetes mellitus; GAPDH: glyceraldehyde-3-phosphate dehydrogenase; GDM: gestational diabetes mellitus; iGDM: insulin-treated gestational diabetes mellitus; IQR: interquartile range; LDLR: low-density lipoprotein receptor; LGA: large for gestational age; LPL: lipoprotein lipase; LRP1: low-density lipoprotein receptor-related protein 1; LRP2: low-density lipoprotein receptor-related protein 2; MTTP: microsomal triglyceride transfer protein; RBP4: retinol binding protein 4; RNA: ribonucleic acid; ROH: retinol; RT-PCR: real-time polymerase chain reaction; RXRα: retinoid X receptor alpha; RXRβ: retinoid X receptor beta; SGA: small for gestational age; STRA6: stimulated by retinoic acid 6; TTR: transthyretin; VLDLR: very low density lipoprotein receptor.
