# Supplementary material for: STRA6 and Placental Retinoid Metabolism in Gestational Diabetes Mellitus
Source: J Pers Med. 2021 Dec 5;11(12):1301. doi: 10.3390/jpm11121301 (PMC8708334; doi:10.3390/jpm11121301)
Supplement: Supplementary file 1 [file jpm-11-01301-s001.zip › jpm-1420758-SI.pdf]

Table S1- Here are listed the primer sequences used.

Primer sequence (5' to 3')	Primer name
TGCTGCTGTTTTCATGGTCC	STRA6 (Hu) FW
TGGAGCCTCAGTTTTCCCAT	STRA6 (Hu) RW
GGCTGATCAGGTGTCGGAAA	LRP1 (Hu) FW
GTCACCGTTGTTGACACTGC	LRP1 (Hu) RW
AGTATGTAACCAGGAGCAGGAC	VLDLR (Hu) FW
CACAGTCACACTCGTAGCCTAT	VLDLR (Hu) RW
CGATGAGTGTCAGGATCCCG	LDLR (Hu) FW
GAAGAGGTAGGCGATGGAGC	LDLR (Hu) RW
CGAGCGCTCCATTCATCTCT	LPL (Hu) FW
CCAGATTGTTGCAGCGGTTC	LPL (Hu) RW
TCTCTACTCGGGTTCTGGCA	MTTP (Hu) FW
AGGTGACAGGTCTGAGCTGA	MTTP (Hu) RW
CTCTGACTTCAACAGCGACAC	GAPDH (Hu) FW
AGCCAAATTCGTTGTCATACCAG	GAPDH (Hu) RW
CTTGCAACTATCCGACCTGC	LRP2 (Hu) FW
GGACCGCTTTCACATCCATC	LRP2 (Hu) RW
CTACGTGGAGGCAAACATGG	RXRα (Hu) FW
CAGCTCTGAGAAGTGTGGGA	RXRα (Hu) RW
ATACTCTTGCCGGGACAACA	RXRβ (Hu) FW
TCCCCATCCTTGTCCTTTCC	RXRβ (Hu) RW
